# A systematic review on laparoscopic subtotal cholecystectomy for difficult gallbladders: a lifesaving bailout or an incomplete operation?

**DOI:** 10.1308/rcsann.2023.0008

**Published:** 2023-06-27

**Authors:** M Al-Azzawi, M Abouelazayem, C Parmar, R Singhal, B Amr, A Martinino, SD Atıcı, K Mahawar

**Affiliations:** ^1^ University Hospital Crosshouse, UK; ^2^ St George’s University Hospitals NHS Foundation Trust, UK; ^3^ Whittington Health NHS Trust, UK; ^4^ University Hospitals Birmingham NHS Foundation Trust, UK; ^5^ University Hospitals Plymouth NHS Trust, UK; ^6^ University of Rome “La Sapienza”, Italy; ^7^ Izmir Tepecik Training and Research Hospital, Turkey; ^8^ South Tyneside and Sunderland NHS Foundation Trust, UK

**Keywords:** Laparoscopic, Gallbladder, Gallstones, Minimally invasive, Subtotal cholecystectomy, Biliary surgery

## Abstract

**Introduction:**

Laparoscopic subtotal cholecystectomy (LSTC) is a bailout procedure that is undertaken when it is not safe to proceed with a laparoscopic total cholecystectomy owing to dense adhesions in Calot’s triangle. The main aim of this review was to investigate the early (≤30 days) and late (>30 days) morbidity and mortality of LSTC.

**Methods:**

A literature search of the PubMed^®^ (MEDLINE^®^), Google Scholar™ and Embase^®^ databases was conducted to identify all studies on LSTC published between 1985 and December 2020. A systematic review was then performed.

**Results:**

Overall, 45 studies involving 2,166 subtotal cholecystectomy patients (51% female) were identified for inclusion in the review. The mean patient age was 55 years (standard deviation: 15 years). Just over half (53%) of the patients had an elective procedure. The conversion rate was 6.2% (*n*=135). The most common indication was acute cholecystitis (49%). Different techniques were used, with the majority having a closed cystic duct/gallbladder stump (71%). The most common closure technique was intracorporeal suturing (53%), followed by endoloop closure (15%). Four patients (0.18%) died within thirty days of surgery. Morbidity within 30 days included bile duct injury (0.23%), bile leak (18%) and intra-abdominal collection (4%). Reoperation was reported in 23 patients (1.2%), most commonly for unresolving intra-abdominal collections and failed endoscopic retrograde cholangiopancreatography to control bile leak. Long-term follow-up was reported in 30 studies, the median follow-up duration being 22 months. Late morbidity included incisional hernias (6%), symptomatic gallstones (4%) and common bile duct stones (2%), with 2% of cases requiring completion of cholecystectomy.

**Conclusions:**

LSTC is an acceptable alternative in patients with a “difficult” Calot’s triangle.

## Introduction

Cholecystectomy is one of the most common abdominal operations performed worldwide.^1^ The operation can be technically difficult owing to dense adhesions in Calot’s triangle. Conversion to open surgery or subtotal cholecystectomy has been described to deal with these situations.^2,3^

Many contemporary surgeons undertaking cholecystectomy may not have expertise in performing this as an open procedure, which has its own challenges due to working in a deep pocket without the illumination and magnification of a laparoscope. Furthermore, conversion to an open procedure may counterintuitively increase the risk of bile duct injuries.^4^ There is therefore a need to evaluate safer options for dealing with a “difficult” Calot’s triangle. In particular, the choice that surgeons face in these situations is whether they should perform a laparoscopic subtotal cholecystectomy (LSTC), which should be possible in most patients, or convert to an open total cholecystectomy.

Subtotal cholecystectomy (STC) is well recognised as an option in patients where a total cholecystectomy may expose the patient to an increased risk of injury to the hepatic pedicle. During this procedure, portions of the gallbladder are left behind when the structures of Calot’s triangle cannot be clearly identified and the critical view of safety cannot be achieved.

A 2021 systematic review and meta-analysis on STC by Nzenwa *et al* suggested high perioperative morbidity associated with the procedure.^5^ These findings are at odds with a previous systematic review and meta-analysis by Elshaer *et al*, published in 2015, which concluded that morbidity rates for STC were comparable with those for total cholecystectomy.^6^ However, both these reviews included patients undergoing open STC (OSTC). This makes it difficult to understand the outcomes of LSTC as surgeons are not generally faced with the choice of converting to open surgery to perform a STC. Instead, the choice is whether to perform LSTC or convert to open surgery for a total cholecystectomy.

For this reason, it is important to establish the outcomes of LSTC alone (without including patients who underwent OSTC). This is particularly relevant during the COVID-19 pandemic as the management of gallstone disease appears to have become more complex.^7,8^

Henneman *et al* published a systematic review on LSTC patients in 2013.^9^ Our aim was to update their review by also including new studies and analysing the subgroup of patients who underwent a completely laparoscopic procedure (i.e. no conversion to open surgery).

## Methods

This study was carried out in accordace with the PRISMA (Preferred Reporting Items for Systematic reviews and Meta-Analyses) guidelines.^10^ The PubMed^®^ (MEDLINE^®^), Google Scholar™ and Embase^®^ databases were searched for all relevant English language articles describing LSTC in human adults (≥18 years) using the keywords “subtotal cholecystectomy”, “gallbladder resection”, “gallbladder excision”, “gallbladder removal”, “partial”, “incomplete”, “insufficient”, “deroofing” and “near-total”. The search was restricted to articles published between 1985 and February 2021 as the first laparoscopic cholecystectomy was described originally in 1985.^11^ Additional articles were also found from references of the papers identified in the intial literature search. The last of these searches was carried out on 21 February 2021.

Case reports, case series with <5 cases, conference abstracts and reviews were excluded. Studies that involved only open cases and those with patients who had a preoperative cholecystostomy were also excluded. Only English language studies were included in our review. A meta-analysis was not undertaken owing to inconsistent reporting of outcome measures, and differences in populations and study design.

### Participants

All studies with five or more cases describing any experience with an adult cohort (≥18 years) of patients undergoing STC while attempting laparoscopic cholecystectomy were included in our review. Studies on patients who underwent preoperative cholecystostomy were excluded. Studies with patients who had LSTC as part of other surgery were also excluded as we wanted to understand the morbidity and mortality of LSTC alone. Studies on patients who underwent OSTC (open from start of procedure) were excluded, as were those where the LSTC cohort was merged with the OSTC cohort and outcomes of LSTC were not reported separately.^12^

Our intention-to-treat analysis comprised all studies on LSTC, including those where some procedures were converted to OSTC. Furthermore, data were analysed separately for studies where no patients were converted to open surgery as the main objective of this review was to understand the outcomes of LSTC as a standalone procedure.

### Study selection and data extraction

**Figure 1:**
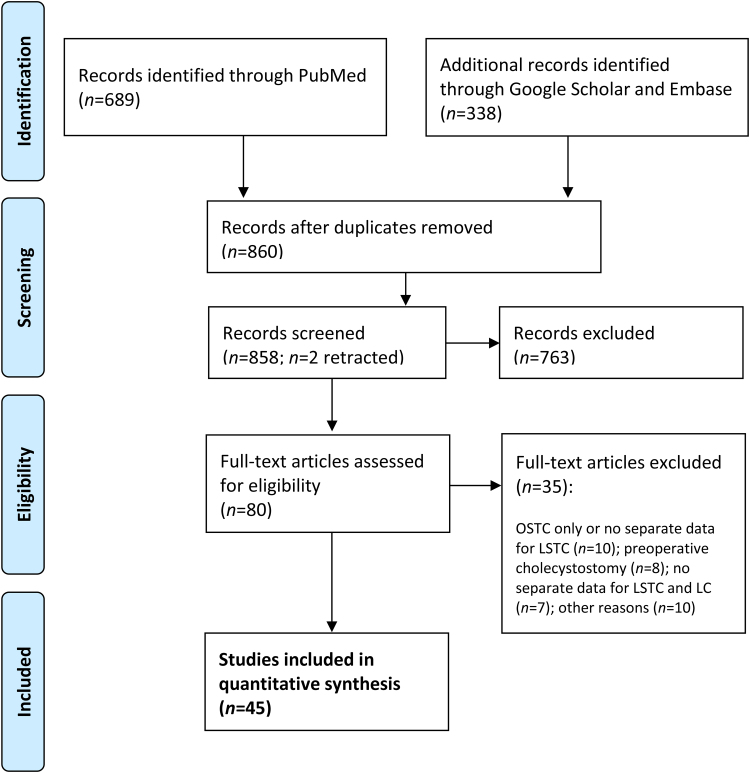
Flowchart of study selection LC = laparoscopic cholecystectomy; LSTC = laparoscopic subtotal cholecystectomy; OSTC = open subtotal cholecystectomy

A PRISMA flowchart summarising the study selection is provided in Figure 1. Two authors (MAZ and MAB) independently screened all the titles identified in the search and discussed any discrepancies. A total of 80 articles were assessed for eligibility by reviewing the full text; this was completed by six of the authors (MAZ, MAB, BA, CP, AM and SDA). MAZ then re-checked all 80 articles, 45 of which were deemed eligible for inclusion in this review. The data were extracted and analysed using Microsoft Excel^®^. When calculating the percentages for variables, different denominators were used as not all variables were reported in every study.

### Study outcomes

The primary outcome measures were early (≤30 days) morbidity and mortality. Secondary outcome measures included conversion to open surgery, length of hospital stay, and late (>30 days) morbidity and mortality.

## Results

The literature search yielded 45 studies with a total of 2,166 STC cases where the procedure started laparoscopically (intention-to-treat analysis).^13–57^ Tables 1 and 2 summarise the patient characteristics and indications. The mean patient age was 55 years (standard deviation [SD]: 15 years). Half of the patients were female (955/1,833, 51%). The mean length of hospital stay was 5.5 days (SD: 4 days). Just over half of the procedures were undertaken as elective cases (390/735, 53%). The conversion rate was 6.2% (*n*=135). The most common cause of conversion to open surgery was dense adhesions (68/135, 50%), followed by severe inflammation or gallbladder perforation and spillage (20/135, 15%), inability to define anatomy (19/135, 14%), intraoperative complications (9/135, 7%) and aberrant anatomy (5/135, 4%). The reason was not mentioned in 14 studies.

**Table 1:** Basic patient demographics and comorbidities for all patients (*n*=2,166)

Mean age*	55 years (standard deviation: 15 years)
*Sex*	
Female	955/1,833 (51%)
Male	878/1,833 (49%)
*Comorbidities*	
Diabetes mellitus	134/394 (34%)
Hypertension	59/227 (26%)
Ischaemic heart disease	39/165 (24%)
Liver cirrhosis	262/724 (36%)
*Data not available for all studies	

**Table 2:** Indications for and urgency of surgery, follow-up duration and length of hospital stay for all patients (*n*=2,166)

*Indications for surgery*	
Acute cholecystitis	763/1,565 (49%)
Chronic cholecystitis	188/649 (29%)
Biliary colic	194/1,434 (14%)
Jaundice	128/1,415 (9%)
Pancreatitis	50/1,412 (4%)
*Urgency of surgery*	
Elective	390/735 (53%)
Emergency	345/735 (47%)
Median follow-up duration*	22 months
Mean length of stay*	5.5 days (standard deviation: 4 days)
*Data not available for all studies	

Thirty of the forty-five studies (including 1,125 patients) described cohorts where not a single case was converted to an open procedure.^13,16,17,19,22,24–28,30,33,35–40,42,44–46,48–50,52–55,57^ This gave us data on outcomes relating solely to LSTC (per-protocol analysis).

### Surgical details

Forty of the studies included in this review described the operative techniques performed.^13–30,32–40,42–45,47–53,55–57^ Half of the patients had the posterior gallbladder wall left in situ (904/1,773, 51%). Different techniques were described for dealing with the cystic duct or gallbladder stump. In the majority of cases, the cystic duct/gallbladder stump was closed (1,188/1,672, 71%). The most common method of closure was intracorporeal sutures (631/1,188, 53%), followed by endoloop (178/1,188, 15%), stapler (80/1,188, 7%), clips (62/1,188, 5%) and a novel technique using an omental plug (40/1,188, 3%). Four studies did not specify how closure was performed (197/1,188, 17%).^13,24,35,56^ The majority of patients had a drain inserted (1,875/2,096, 89%). Table 3 summarises the technical details for all patients.

**Table 3:** Technical details for all patients (*n*=2,166)

*Technique*	
Posterior wall in situ	904/1,773 (51%)
Removal of posterior wall	869/1,773 (49%)
Closure of cystic duct/gallbladder stump	1,188/1,672 (71%)
Non-closure of cystic duct/gallbladder stump	484/1,672 (29%)
*Closure of cystic duct or gallbladder stump*	
Sutures	631/1,188 (53%)
Endoloops	178/1,188 (15%)
Staples	80/1,188 (7%)
Clips	62/1,188 (5%)
Omental plug	40/1,188 (3%)
Not specified	197/1,188 (17%)
Drain insertion	1,875/2,096 (89%)
Intraoperative complications	14/2,166 (0.6%)
Bleeding	5
Bile duct injury	4
Bowel injury	3
Liver injury	2
Conversion to open surgery	135/2,166 (6.2%)

The different surgical techniques were not analysed separately. This was owing to the fact that not all papers made it clear why certain techniques were adopted over others; this could be due to intraoperative findings or the surgeon’s preference.

#### Early morbidity and mortality

Intraoperative complications were reported in 0.6% of patients (*n*=14). These included bleeding (*n*=5), bile duct injury (*n*=4), bowel injury (*n*=3) and liver injury (*n*=2).

Four deaths occurred within 30 days of surgery (0.18%). One patient died from postoperative pulmonary embolism, one developed postoperative myocardial infarction, and one was immunosuppressed, and developed pulmonary sepsis and multiorgan failure. The fourth patient, who was elderly and had chronic liver disease with portal hypertension and a gangrenous gallbladder, developed acute respiratory distress syndrome with decompensated chronic liver disease.

Five patients overall (0.23%) were noted to have a bile duct injury (4 detected intraoperatively and 1 postoperatively). The bile duct was repaired laparoscopically as a primary repair in two cases, one case was converted to an open procedure and the bile duct repaired with a T-tube, and in one case, the bile duct injury was diagnosed postoperatively and the patient underwent a hepaticojejunostomy several weeks later. There was no information on how the bile duct injury was managed for the fifth case.

The most common early complication was bile leak (383/2,166, 18%). Other early complications included intra-abdominal collection (77/1,751, 4%) and wound infection (55/1,239, 4%). Chest infection was reported in 4% of patients (17/396). Six per cent of patients (116/1,815) required endoscopic retrograde cholangiopancreatography (ERCP), the majority being for bile leak (*n*=59). It was not specified in the majority of papers whether this was for a persistent minor leak or a major leak. There were 23 reoperations (1.2%); the indications were: intra-abdominal collection not resolving with antibiotics/drain not functioning (*n*=14), failed ERCP for bile leak (*n*=4) and bleeding from liver edge (*n*=1). For four patients, the indication for reoperation was not specified.

#### Late morbidity and mortality

Thirty studies (1,418 patients) reported on long-term follow-up.^13–15,17,18,20,24,26–30,32,34–37,40–47,50,51,53,54,56^ The median follow-up period was 22 months. Four deaths occurred in the period after thirty days following surgery. The most common late morbidity was incisional hernia (22/352, 6%), followed by symptomatic gallstones (41/980, 4%) and common bile duct (CBD) stones (25/1,096, 2%). Completion cholecystectomy was needed in 2% of patients (22/1,267). Late (>30 days) ERCP was required in 3% of patients (29/1,003), mostly for CBD stones.

Table 4 summarises the early and late postoperative outcomes and interventions.

**Table 4:** Postoperative outcomes and interventions for all patients (*n*=2,166)

*Early outcomes (*≤*30 days)*	
Bleeding	6/1,666 (0.36%)
Bile duct injury (intra and postoperative)	5/2,166 (0.23%)
Bile leak	383/2,166 (18%)
Intra-abdominal collection	77/1,751 (4%)
Wound infection	55/1,239 (4%)
Chest infection	17/396 (4%)
Mortality	4/2,166 (0.18%)
*Early interventions (*≤*30 days)*	
Endoscopic retrograde cholangiopancreatography	116/1,815 (6%)
Reoperation	23/1,956 (1.2%)
*Late outcomes (>30 days)*	
Incisional hernia	22/352 (6%)
Symptomatic gallstones	41/1,040 (4%)
Common bile duct stones	25/1,096 (2%)
Endoscopic retrograde cholangiopancreatography	29/1,003 (3%)
Completion cholecystectomy	22/1,267 (2%)
Mortality	4/1,444 (0.28%)

### Subgroup analysis (LSTC)

Thirty studies (involving 1,125 patients) described cohorts where all the procedures were completed laparoscopically (i.e. no cases were converted to open surgery).^13,16,17,19,22,24–28,30,33,35–40,42,44–46,48–50,52–55,57^ In this subgroup, 52% of the patients were women. A third (29%) of the patients had diabetes and a fifith (22%) had hypertension. As expected, the most common indication for LSTC was acute cholecystitis (51%), followed by chronic cholecystitis (25%), jaundice (9%) and biliary colic (7%); only 2% had their procedure for acute pancreatitis.

The gallbladder stump was closed in 72% of the LSTC cohort. The vast majority (94%) of patients had a drain left in situ.

#### Early morbidity and mortality in LSTC subgroup

The incidence of intraoperative complications in the LSTC subgroup was 0.8%. These comprised bleeding (*n*=4), bile duct injury (*n*=2), liver injury (*n*=2) and bowel injury (*n*=1). Two patients died within 30 days of surgery.

Fourteen per cent of the LSTC group had a bile leak. Other complications included chest infection (8%), wound infection (4%) and intra-abdominal collection (4%). There were only two cases of bile duct injury, giving an incidence of 0.18%.

#### Late morbidity and mortality in LSTC subgroup

Eighteen of the thirty studies with data available for LSTC subgroup analysis (involving 669 patients) reported on long-term follow-up.^13,17,24,26–28,30,35–37,40,42,44–46,50,53,54^ These studies showed a 2% risk of incisional hernia, a 2% risk of ERCP and a 1% risk of symptomatic gallstones.

The data for the LSTC subgroup analysis are summarised in Table 5.

**Table 5:** Postoperative outcomes and interventions for laparoscopic subtotal cholecystectomy patients only (per-protocol analysis) (*n*=1,125)

*Early outcomes (*≤*30 days)*	
Bleeding	5/930 (0.54%)
Bile duct injury (intra and postoperative)	2/1,125 (0.18%)
Bile leak	152/1,125 (14%)
Intra-abdominal collection	47/1,068 (4%)
Wound infection	21/589 (4%)
Chest infection	14/182 (8%)
Mortality	2/1,125 (0.18%)
*Early interventions (*≤*30 days)*	
Endoscopic retrograde cholangiopancreatography	71/845 (8%)
Reoperation	14/995 (1.4%)
*Late outcomes (>30 days)*	
Incisional hernia	4/247 (2%)
Symptomatic gallstones	7/487 (1%)
Endoscopic retrograde cholangiopancreatography	9/365 (2%)
Completion cholecystectomy	4/547 (1%)
Mortality	0/669 (0%)

## Discussion

This paper presents an up-to-date review on LSTC. Henneman *et al* published a systematic review on LSTC in 2013 but our review includes data from many more patients (2,166 vs 625)*.*^9^ Our findings indicate that STC is associated with a high rate of bile leak (18%) although it was not possible to discern how many of these cases were simply minor leaks. There was a low rate of bile duct injury (0.23%). Late morbidity rates also appear to be low, with the incidence of symptomatic gallstones being only 4%. Our review included a subanalysis of those studies where all patients underwent LSTC without the need for conversion to OSTC.

Erich Mühe performed the first laparoscopic cholecystectomy on 12 September 1985 in Germany^11,58^ but it was not until the 1990s that the laparoscopic approach became the gold standard for cholecystectomy.^59,60^ It is now among the most commonly performed elective day case procedures.^61^ Nevertheless, one of the major complications is bile duct injury, a catastrophic complication^62,63^ that can even reduce long-term survival.^1^ Injuries to the hepatic pedicle are more common in patients with dense adhesions in Calot’s triangle. In these patients with a difficult Calot’s triangle, LSTC has become more popular as an alternative option in cases where previously a conversion to open cholecystectomy would have been performed.^19–21,24,64^ The laparoscopic approach offers the advantage of better illumination and magnification in addition to reduced pain and other complications associated with open surgery, and STC allows for control of the disease process without putting patients at undue risk.

Surgeons use different techniques for LSTC. Some studies describe removal of both the anterior and posterior walls, and some describe removal of the anterior wall only. Some surgeons close the gallbladder stump, using different methods (intracorporeal sutures, endoloop, clips, stapler or omental plug), and some leave it open. In our view, the key objectives of LSTC are to remove all impacted stones from Hartmann’s pouch and to control bile flow, which should preferably be achieved by closing the cystic duct orifice through the lumen of the gallbladder. Closure of Hartmann’s pouch with an endoloop or suture are other options but these may be associated with remnant cholecystitis postoperatively, especially if a large remnant is left.^65,66^

One proposed classification by Henneman *et al* described four types of LSTC depending on whether the posterior wall was left in situ and whether the gallbladder remnant was closed or left open.^9^ For types A and B, the posterior wall is left in situ, with the remnant being closed in type B, whereas for types C and D, the posterior wall is removed, with the remnant being closed in type C.

Elshaer *et al* published a systematic review on STC in 2015, concluding that it was an important tool in treating difficult gallbladders.^6^ However, their review included both OSTC and LSTC. Conversely, given that most surgeons convert from LSTC to open surgery not to perform an OSTC but to perform a total cholecystectomy, our review sought to understand the outcomes of LSTC alone.

The risk of bile duct injury in cholecystectomy appears to be higher for the laparoscopic approach (0.4–0.6%) than for the open approach (0.1–0.2%).^67^ A bile duct injury rate of 0.23% (*n*=5) was seen in our review for STC, which compares well with these figures, once again highlighting the safety of this approach. Our rate is also similar to those reported by Henneman *et al* (0.16%),^9^ Elshaer *et al* (0.08%)^6^ and Nzenwa *et al* (0.2%)^5^ although it should be noted that the reviews by Elshaer *et al* and Nzenwa *et al* also included STC patients where surgery was open to start with.

One of the major morbidities in LSTC is bile leak. In our review, the incidence of bile leak was 18% but only 6% of patients required postoperative ERCP for a persistent leak. This is the same as the rate reported by Elshaer *et al* for bile leak^6^ but a lower rate of 10.6% was noted in the systematic review by Henneman *et al*.^9^ Bile leak is one of the main drawbacks of LSTC and this may be due to the difficulty in performing intracorporeal suturing. Surgeons use several different techniques to close the cystic duct/gallbladder stump. As expected, the risk of bile leak was higher in patients where the gallbladder remnant/cystic duct was left open; It was 15–72% for these patients^16,18,38,44,52,53^ compared with 0–65% for those where the gallbladder remnant/cystic duct was closed.^13,14,17,20,22,23,25,26,28,30,32–37,39,40,42,43,45,47,51,55^

Our review showed a 1.2% reoperation rate (*n*=23). The majority of these cases were secondary to unresolved bile leak/abdominal collection. Elshaer *et al* noted that 1.8% of patients underwent reoperation, with a quarter of these requiring CBD exploration.^6^ They also reported an ERCP rate of 4.1%, mostly for retained CBD stones or bile leak. The ERCP rate calculated from our data was 6%, most of these cases being performed for bile leak.

One of the concerns regarding STC is that patients can have recurring symptoms secondary to the gallbladder remnant. In this review, 4% of patients (*n*=41) reported symptoms, with 2% (*n*=22) undergoing a completion cholecystectomy. This compared with 2.2% of patients experiencing recurrent symptoms in the review by Henneman *et al.*^9^ This difference may be due to the closure of Hartmann’s pouch. The problem can be effectively tackled by leaving Hartmann’s pouch open and suturing the cystic duct orifice if it can be visualised.

The studies included in our review reported a total of eight mortalities (0.4%); there were four deaths within 30 days of surgery and four deaths after 30 days. The four mortalities during long-term follow-up were due to unrelated conditions.

This systematic review on LSTC updates a previous review published in 2013 by Henneman *et al*.^9^ Compared with the 15 studies and 625 patients in that earlier review, our review includes 45 studies with a total of 2,166 patients. We have also separately analysed outcomes in the LSTC subgroup. As such, our review provides a comprehensive overview of what patients can expect from a LSTC. As recommended by the Prevention of Bile Duct Injury Consensus Work Group in 2020, when the critical view of safety is not obtained, then performing a LSTC is safer than attempting a total cholecystectomy.^68^ The results for long-term outcomes would be beneficial when consenting patients.

### Study limitations

One of the limitations of this review is the exclusion of papers not published in English. The review was also constrained by the quality of the included studies. There are no randomised studies on this topic. The studies that do exist had variable outcome measures and inconsistent reporting. Nevertheless, the data are still able to provide reasonable estimates for early as well as late morbidity and mortality of LSTC.

## Conclusions

LSTC is an acceptable alternative in patients with a “difficult” Calot’s triangle. Although LSTC can be associated with bile leak (which can mostly be managed non-operatively), this would still outweigh the risk of sustaining a bile duct injury when attempting a total cholecystectomy in a difficult operative field.
